# The frequency of assessment tools in arthroscopic training: a systematic review

**DOI:** 10.1080/07853890.2022.2085317

**Published:** 2022-06-13

**Authors:** Haixia Zhou, Chengyao Xian, Kai-Jun Zhang, Zhouwen Yang, Wei Li, Jing Tian

**Affiliations:** a The Second School of Clinical Medicine, Southern Medical University, Guangzhou, China; b Department of Clinical Skills Training Center, Zhujiang Hospital, Southern Medical University, Guangzhou, China; c Department of Orthopedics, Zhujiang Hospital, Southern Medical University, Guangzhou, China

**Keywords:** Arthroscopy, training, assessment, arthroscopic skills, feedback

## Abstract

**Background:**

Multiple assessment tools are used in arthroscopic training and play an important role in feedback. However, it is not fully recognized as to the standard way to apply these tools. Our study aimed to investigate the use of assessment tools in arthroscopic training and determine whether there is an optimal way to apply various assessment tools in arthroscopic training.

**Methods:**

A search was performed using PubMed, Embase and Cochrane Library electronic databases for articles published in English from January 2000 to July 2021. Eligible for inclusion were primary research articles related to using assessment tools for the evaluation of arthroscopic skills and training environments. Studies that focussed only on therapeutic cases, did not report outcome measures of technical skills, or did not mention arthroscopic skills training were excluded.

**Results:**

A total of 28 studies were included for review. Multiple assessment tools were used in arthroscopic training. The most common objective metric was completion time, reported in 21 studies. Technical parameters based on simulator or external equipment, such as instrument path length, hand movement, visual parameters and injury, were also widely used. Subjective assessment tools included checklists and global rating scales (GRS). Among these, the most commonly used GRS was the Arthroscopic Surgical Skill Evaluation Tool (ASSET). Most of the studies combined objective metrics and subjective assessment scales in the evaluation of arthroscopic skill training

**Conclusions:**

Overall, both subjective and objective assessment tools can be used as feedback for basic arthroscopic skill training, but there are still differences in the frequency of application in different contexts. Despite this, combined use of subjective and objective assessment tools can be applied to more situations and skills and can be the optimal way for assessment.

**Level of Evidence:**

Level III, systematic review of level I to III studies.

**Key messages**
- Both subjective and objective assessment tools can be used as feedback for basic arthroscopic skill training.
- Combined use of subjective and objective assessment tools can be applied to more situations and skills and can be the optimal way for assessment.

## Introduction

Arthroscopy is one of the common diagnostic and therapeutic tools in orthopaedics [1,2]. Because of its indispensable role in orthopaedic diagnosis and surgery, more attention has been paid to formulating a structured arthroscopic training program [3]. In fact, assessment and training are synergistic, as assessing trainees is essential to ensure appropriate learning of skills and to identify deficiencies [4–6]. In response, multiple assessment tools have been developed specific to arthroscopic training in both simulated and clinical environments that can be classified as objective and subjective tools.

Objective assessment tools depend on easy-to-measure metrics. The direct statistics can provide an accurate result of procedures without subjective interference. Currently, due to the advent of simulator models used for arthroscopic training [7], more technical parameters based on the simulator built-in systems or external equipment are considered as common assessment tools, such as motion analysis [8–10], force patterns (haptic) [11,12], and visual parameters [13]. The installed software converts the movement and positional data generated by sensors to assessment of motor dexterity and visuospatial ability [4,13]. These sensitive technical metrics can discriminate among various levels of arthroscopic skills and allow objective measurements of trainees’ performances.

Subjective assessment scales commonly take the form of scoring and have predetermined criteria that reduce the element of subjectivity to make the evaluation more reliable [14]. Global rating scales (GRS), as common subjective assessment tools, contain several constituent domains [15,16]. For example, the Arthroscopic Surgery Skill Evaluation Tool (ASSET) global rating scale consists of nine domains, including a weighted descriptor, “additional complexity of procedure”, as a control measure for special skills. Each domain discriminate novice, competent and expert level with a 5-point Likert-type scale. Subjective assessment scales have been reported in previous studies in both simulated and clinical environments and have been recognized as practical and feasible assessment tools [14].

Although various assessment tools are increasingly used in arthroscopic skills training and previous studies have determined that there is sufficient evidence for the use of assessment tools in arthroscopic training, some are limited to specific assessment contexts [15,17]. For instance, parameters generated by sensors may be more limited in the simulated environment. In addition, consistent outcome reporting after training is necessary, but there is considerable heterogeneity in different assessment settings [18]. Cost effectiveness is also one of the factors to consider when choosing an evaluation tool, given the expense of additional equipment such as sensors.

Inconsistent use of assessment tools interferes with the discrimination of skill levels and the assessment of training outcomes; however, few comparative studies of tools are available. Clarifying the usage scenarios and practicability of different assessment tools can provide better training of arthroscopic skills.

This systematic review aimed to investigate the use of assessment tools in arthroscopic training and determine whether there is an optimal way to apply various assessment tools in arthroscopic training. It is hypothesized that both subjective and objective assessment tools can be used as feedback for basic arthroscopic skill training and that their combined use can be the optimal way for assessment.

## Methods

### Study eligibility

Eligible for inclusion were primary research studies related to using assessment tools to evaluate arthroscopic skills training in simulated or clinical environments. Language was limited to English. Studies that focussed only on therapeutic care, did not report outcome measures of technical skills, or did not mention arthroscopic skills training were excluded. Non-English language articles, reviews and conference abstracts were also excluded. This systematic review complied with the Preferred Reporting Items of Systematic Reviews and Meta-Analyses (PRISMA) guidelines [19].

### Literature search

We performed a literature search using PubMed (all fields), Embase (all fields) and Cochrane Library (all text) electronic databases. Articles related to arthroscopic skills published in English from 2000 to July 2021 were included for screening. The search strategy was as follows: (training OR learning OR education OR assessment OR evaluation) AND (technical OR competence OR skill) AND (arthroscopy OR arthroscopic).

Duplicate studies were deleted, titles and abstracts of search results were screened for initial eligibility, and retrieved full articles were evaluated by two reviewers. The reference lists were screened to identify and retrieve other relevant studies.

### Risk of bias assessment

The study quality assessment tools developed by the National Heart, Lung and Blood Institute were used to assess the risk of bias in the included studies [20]. The Quality Assessment of Controlled Intervention Studies (Appendix 1) was used to grade randomized controlled studies, and the Quality Assessment Tool for Before-After (Pre-Post) Studies With No Control Group (Appendix 2) was used to assess noncontrolled studies. In general terms, a "good" study has the least risk of bias, and results are considered to be valid. A "fair" study is susceptible to some bias but deemed not sufficient to invalidate its results. A "poor" rating indicates significant risk of bias.

### Data abstraction and data analysis

From eligible studies, extracted data included authors, date of publication, participants, joints, testing context, skills assessed, and objective metrics or subjective tools used to assess arthroscopic skills. The details of reported measurement outcomes in each study are summarized in the tables.

The primary outcome measure was the type of assessment tools or metrics used in the studies; these can be classified into subjective and objective, based on the type of method. Frequency and testing environments were also included to analyse the usage of assessment tools or metrics.

## Results

### Study selection

**Figure 1.**
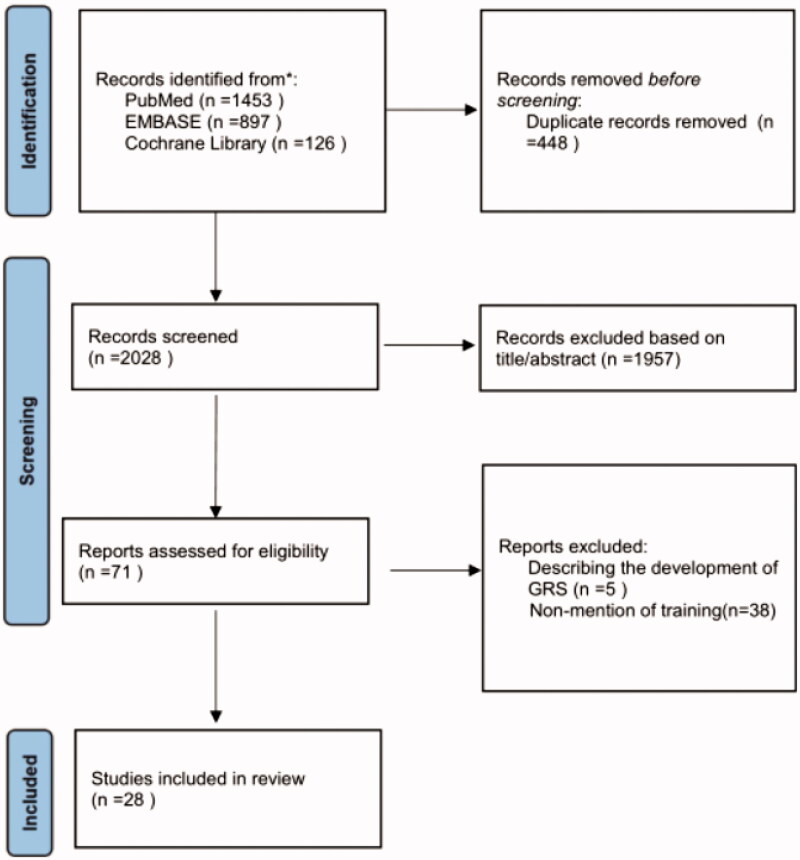
PRISMA flow diagram of study selection process.

From the search, 71 studies were selected after screening of titles and abstracts. After full-text eligibility assessment, five studies describing the development and content of different global rating scales were excluded. There were 38 studies that did not mention arthroscopic skills training and were therefore excluded. Thus, after full-text screening, 28 studies satisfied the inclusion criteria and were included for qualitative analysis (Figure 1).

### Risk of bias assessment

In total, 24 studies were assessed by the Quality Assessment of Controlled Intervention Studies, and the other four studies were assessed using the Quality Assessment Tool for Before-After (Pre-Post) Studies with No Control Group. Nineteen of the 28 studies were judged to be “good” and 9 of the 28 studies were judged to be of “fair.” There was no study considered as “poor.” Therefore, all studies selected were considered eligible.

### Study characteristics

The descriptive characteristics of the included studies are listed in Table 1. The majority of arthroscopic skills testing was performed in simulated environments, including a simulator [21–38], cadaver [33,37,39–44] and animal model [45], and five studies [32,38,46–48] were performed on patients. Of the included studies, participants mainly involved medical students, orthopaedic residents, surgeons and experts. In one study, the participants were not specified. Among types of arthroscopic skills assessed in the studies, the majority (20 studies) concerned diagnostic arthroscopy as all or part of the testing task; other tasks included triangulation (six studies), removing loose bodies (three studies), probing examination (two studies), meniscectomy (three studies) and anterior labral repair (one study).

**Table 1.** Summary of included studies.

Study	Participants	Joint	Context	Skills Assessed	Objective Metrics	Subjective Tools	QA	LOE
Ferguson et al., 2017 [21]	18 medical students	Knee and shoulder	Simulator	Diagnostic arthroscopy	Task completion time; path length of subject’s hands; number of hand movements	BAKSSS	Fair	2
Li *et al*, 2020 [22]	16 residents	Knee	Simulator	Remove loose bodies; triangulation; guided diagnostic	Task completion time; camera path length; cartilage injury	–	Good	1
Alvand et al., 2011 [24]	33 medical students	Knee and shoulder	Simulator	Triangulation; remove loose bodies	Task completion time; path length of subject’s hands; number of hand movements	–	Fair	2
Andersen et al., 2011 [25]	14 surgeons	Shoulder	Simulator	Diagnostic arthroscopy	Task completion time; camera and probe path length; depth and number of collisions	–	Good	2
Beaudoin et al., 2021 [26]	30 medical students	Knee	Simulator	Diagnostic arthroscopy; meniscectomy	Task completion time; camera path length; meniscus cutting score visual parameters;	OAAS; CBA	Good	2
Wang et al., 2019 [37]	28 medical students	Knee and shoulder	Simulator and cadaver	Diagnostic arthroscopy	Task completion rate	ASSET	Good	1
Bhashyam et al., 2017 [27]	48 surgeons and residents	–	Simulator	Triangulation	Highest tapped number; number of errors; visualization loss; number of lookdowns	–	Good	2
Camp et al., 2016 [39]	45 residents	Knee	Cadaver	Diagnostic arthroscopy	Task completion time	ASSET	Good	2
Cychosz et al., 2018 [28]	43 medical students	Knee	Simulator	Diagnostic arthroscopy	Task completion time; camera path length; cartilage damage	–	Fair	2
Dunn et al., 2015 [46]	17 residents	Shoulder	Patient	Diagnostic arthroscopy	Task completion time	14-point diagnostic shoulder arthroscopy checklist; ASSET	Good	2
Henn et al., 2013 [41]	17 medical students	Shoulder	Cadaver	Probing examination	Task completion time	GOALS	Fair	2
Frank et al., 2019 [29]	36 subjects	–	Simulator	Triangulation	Task completion time; task completion rate	–	Good	2
Hauschild *et al*, 2021 [40]	38 residents	Shoulder	Cadaver	Anterior labral repair	–	Procedural step checklist; ASSET	Good	2
Garfjeld Roberts et al., 2019 [47]	30 residents	Knee	Patient	Diagnostic arthroscopy	Task completion time; number of hand movements; smoothness of movements	–	Good	2
Howells et al., 2008 [48]	20 junior trainees	Knee	Patient	diagnostic arthroscopy	–	OCAP checklist; OSATS	Good	2
Huri et al., 2021 [30]	34 orthopaedics trainees	Shoulder	Simulator	Diagnostic arthroscopy; remove loose bodies	Task completion time; camera and grasper path length; scratching of cartilage surfaces	–	Fair	2
Jackson et al., 2012 [31]	19 residents	Knee	Simulator	Meniscal repair	Task completion time; path length of subject’s hands; number of hand movements	–	Good	2
Kim et al., 2017 [45]	14 residents	Knee	Porcine knee model	Diagnostic arthroscopy	Task completion time	ASSET	Fair	3
Martin et al., 2016 [34]	48 residents	Shoulder	Simulator	Diagnostic Arthroscopy	Task completion time; probe distance and tip distance;	–	Fair	3
Ledermann et al., 2020 [32]	11 residents	Knee	Simulator and patient	Meniscectomy	–	ASSET	Good	2
Bouaicha et al., 2020 [23]	18 medical students, 1 intern	Knee	Simulator	Triangulation	Task completion time; camera path length	–	Good	2
Martin et al., 2015 [33]	29 interns and residents	Ankle	simulator and cadaver	Diagnostic arthroscopy	Task completion time	15-point diagnostic ankle arthroscopy checklist; ASSET	Good	1
Middleton et al., 2016 [35]	17 medical students or interns	Knee	Simulator	Diagnostic arthroscopy	Task completion time; number of hand movements	BAKSSS	Good	2
Rahm et al., 2018 [36]	20 residents and 5 experts	Knee and shoulder	Simulator	Diagnostic arthroscopy probing examination	Task completion time; camera path length	ASSET	Fair	3
Redondo et al., 2020 [43]	28 medical students	Knee and shoulder	Cadaver	Diagnostic arthroscopy; triangulation	Task completion rate	ASSET	Good	2
Sandberg et al., 2017 [44]	24 medical students	Knee	Cadaver	Diagnostic arthroscopy	Number of attempts to reach proficiency	BAKSSS	Good	2
Rebolledo et al., 2015 [42]	14 residents	Knee and shoulder	Cadaver	Diagnostic arthroscopy	Task completion time	Injury grading index (IGI)	Fair	2
Waterman et al., 2016 [38]	22 residents	Shoulder	Simulator and patient	Diagnostic arthroscopy	Simulator: completion time; camera and probe distance Patient: completion time	Patient: 14-point diagnostic arthroscopy checklist; ASSET	Good	2

Note: QA: quality assurance; LOE: level of evidence.

### Assessment tools used in studies

A variety of objective metrics was used in the included studies. The most common measurement outcome was completion time, reported in 21 studies (75.0%). Technical parameters based on simulators or external equipment were also widely used in eligible studies. Instrument path length was reported in nine studies (32.1%). Hand movement was reported in five studies (17.9%). Visual parameters such as prevalence of instrument loss were reported in two studies (7.1%). Collisions and injuries were described in five studies (17.9%). Five studies (17.9%) identified individual procedural metrics, such as number of errors, number of attempts, and task completion rate. Objective metrics used in studies are summarized in Table 2.

**Table 2.** Objective outcome metrics used in studies.

Measurement outcomes	No. of studies	%
Completion time	21	75.0
Instrument path length	9	32.1
Hand movement	5	17.9
Visual parameters	2	7.1
Collisions and injuries	5	17.9
^a^Individual procedural metrics	5	17.9

^a^Number of errors, number of attempts, task completion rate.

Subjective tools used for assessing the arthroscopic skills include task-specific checklists and GRS. To score the performance of participants, in total there were four types of checklists from five studies: on the knee (one study) [48], shoulder (three studies) [38,40,46] and ankle (one study) [33]. Six types of GRS were reported in the eligible studies, and an Injury Grading Index Performance Scale (IGI)[42] fulfilled the inclusion criteria. The most commonly used GRS for assessment was the Arthroscopic Surgical Skill Evaluation Tool (10 studies) [32,33,36–40,43,45,46], followed by Basic Arthroscopy Knee Skill Scoring System (three studies) [21,35,44] and the other GRS were reported only in one study each [26,33,41,48]. Descriptions of studies using checklists or GRS are summarized in Table 3.

**Table 3.** Subjective assessment tools used in studies.

Assessment Tools	Description	No. of Studies
Checklist	14-point diagnostic shoulder arthroscopy checklist	2
	Task-specific checklist of anterior shoulder stabilization	1
	Orthopaedic Competence Assessment Project (OCAP) procedure-based assessment for diagnostic arthroscopy	1
	15-point diagnostic ankle arthroscopy checklist	1
GRS	Arthroscopic Surgical Skill Evaluation Tool (ASSET)	10
	Basic Arthroscopy Knee Skill Scoring System (BAKSSS)	3
	Objective Assessment of Arthroscopic Skill (OAAS)	1
	Modified Global Operative Assessment of Laparoscopic Skills (GOALS)	1
	Modified Objective Structured Assessment of Technical Skill (OSATS)	1
	Modified Competency-Based Assessment Form (CBA)	1
	Injury Grading Index Performance Scale (IGI)	1

### Testing context of outcome assessment

Table 4 displays the application of objective and subjective assessment tools in different contexts. In simulated environments, objective metrics were more frequently used than subjective tools. Among the objective indicators in clinical environments, only completion times and hand movements were reported [38,46,47], but all studies related to patients used GRS or checklists. In addition, most of studies (60.7%) combined objective metrics with subjective assessment scales in evaluation of arthroscopic skill training.

**Table 4.** Assessment tools or metrics in testing context.

Testing context	Subjective tools		Objective metrics				
	GRS	Checklist	Time	Length	Movement	Injury	Visual
Simulator (18)	7	1	16	9	4	5	2
Cadaver (8)	8	2	5	0	0	0	0
Animal model (1)	1	0	1	0	0	0	0
Patient (5)	4	3	3	0	1	0	0

The types of simulators included bench-top, VR, and box arthroscopy trainer.

Time = Completion time; Length = Instrument path length; Movement = Hand movement; Injuries = Collisions and injuries; Visual = Visual parameters.

## Discussion

Comprehensive and accurate assessment of surgical competence is essential for arthroscopic skills training, and it can be done using objective and subjective assessment tools. Our review found that objective assessment metrics were used in majority of eligible studies, and these can be classified into completion time, instrument path length, hand movements, visual parameters and injury. Among them, completion time was the most widely used metric. The subjective assessment tools were reported in 17 studies, including four types of checklists and seven GRS. The most commonly used GRS for arthroscopic training was ASSET, followed by BAKSS. Although objective and subjective assessment tools were widely used in both simulated and clinical environments, there were still preferences. In terms of the frequency of use, objective metrics were more commonly used in simulated environments, while GRS are used more with actual patients.

### Objective assessment metrics used in simulated and clinical environment

Twenty-five of the 28 studies used objective metrics, indicating their wide use in arthroscopic skills training. Completion time was most commonly reported and is easy to measure in both simulated and clinical environments. Several studies have shown a significant difference in task completion time compared with the baseline after training [29,46], and this metric can discriminate between different arthroscopic skill levels [49–51]. Furthermore, measuring motion parameters based on the simulation built-in scoring system or external equipment, such as instrument path length and hand movements, is also promising for assessment. Howells et al. [8] demonstrated the validity of a motion analysis system as a means of objective assessment of arthroscopic skills in performing simple tasks. Other objective parameters, such as visual parameters [13,49], collision and injury [52] also showed utility in the simulated environment.

Although objective metrics are convenient and their evidence is reliable, they are inevitably restricted to the environment of use. For example, most motion analysis parameters are derived from the simulator itself or external sensors, which makes their use limited to only simulated environments and not to real patients [53]. Currently, the study of motion analysis is confined to basic arthroscopy tasks [8]. It is not clear whether improvements in these parameters translate into improvements in operating room performance [2]. The same parameters may also vary with different simulators. Middleton et al. [35] found that there was no difference in objective performance between virtual reality (VR) trained and bench-top trained subjects on the final VR simulator wireless objective motion analysis assessment, but a significant difference was seen in the GRS. They proposed that this may be due to the VR simulator itself, in that the shortest path is a function of the physical dimensions of the simulator.

In addition, objective evaluation tools may not always accurately reflect the operator’s skill level. Although completion time is the most commonly used metric, we need to ensure it accurately represents the true skill level, especially in clinical environments, and affirm that speed is not exactly equivalent to proficiency [53]. In a real operating room, there are many factors that affect the operating time, such as teamwork, decision making and communication [15]. In addition, Kim et al. [45] used time as a metric to evaluate arthroscopy skills training on porcine knees. They found that there were no statistically significant differences in time in the fellow groups, whereas it was significant in the junior and senior resident groups, indicating that measuring time is significant only in those with less experience. Alvand et al. [24] found similar results. Based on motion analysis parameters, the training group performed better on the shoulder task but there was no significant difference on the knee task, which is not what they expected. These findings suggest that objective evaluation tools alone may not be the gold standard for assessing skill level but can be used as an effective auxiliary tool.

Assessment and training are synergistic, so a valid assessment should play a meaningful role in guiding training and providing specific remedial measures [4]. However, objective metrics cannot identify weaknesses in specific skills, so the assessment cannot provide targeted training strategies. Although flawed, objective metrics are still the most widely used evaluation method, especially in simulated environments.

### Subjective assessment tools used in simulated and clinical environment

Checklists and GRS were commonly used subjective assessment tools in the studies reviewed. The checklist allows evaluation of whether a key procedure of a task has or has not been performed [54]. It has been said that it turns examiners into observers of behaviour rather than interpreters of behaviour, thereby removing subjectivity in the evaluation process [55]. However, Regehr et al. [55] showed that compared with checklists, GRS scored by experts showed higher inter-station reliability and better construct and concurrent validity.

Among the seven GRS included, three were specially designed for evaluating arthroscopic skills and other the three were modified from assessment scales for different surgical skills. An Injury Grading Index Performance Scale (IGI) fulfilled the inclusion criteria as well, which was designed to subjectively evaluate potential intra-articular injury [42]. Most previous studies have demonstrated that the content, concurrent and construct validity, the reliability of GRS, and current evidence are sufficient to support the use of GRS as a feedback tool under controlled conditions [15]. For example, Koehler et al. [56] tested the validity and reliability of the ASSET as a pass–fail examination of arthroscopic skills, evaluating the participants’ performances on diagnostic knee arthroscopy on a cadaver specimen. Participants passed the test if they attained a minimum score of 3 in each of the eight domains. The likelihood of achieving a passing score on the ASSET increased as postgraduate training increased, and there was considerable agreement between raters as well, thus supporting their hypotheses.

Although GRS are widely used, their effectiveness in the clinical environment is not established. Most of the studies assessed arthroscopic training in simulated environments, and few studies evaluated the validity and reliability of GRS in actual patients, so sufficient evidence to support the applicability of GRS in clinical environments is lacking. In Howells et al.’s [48] study, despite their finding further differences between the simulator-trained group and an untrained group in the operating theatre using the OCAP checklist and a modified OSATS, the result did not confirm the reliability of GRS. Koehler et al. [57] verified the validity and reliability of using the ASSET to assess arthroscopic skill in the operating room. Although a substantial inter-rater reliability was found for diagnostic arthroscopy, rater agreement varied on individual ASSET domains, especially on difficulty of procedure. In addition, they did not assess intra-observer agreement for each rater in the study. Furthermore, according to previous studies, GRS are mainly used to evaluate diagnostic arthroscopy but rarely used for therapeutic procedures. Since therapeutic arthroscopic procedures are common in clinical settings, further study is necessary to determine the utility of GRS in arthroscopy in patients having procedures that are more complex.

Additionally, there is no evidence to confirm the criteria score using GRS to validate trainees’ competency levels. As the most frequently used assessment tool, ASSET did not have exactly the same criteria for identifying minimum competency in different studies. Koehler et al. [58], who developed the ASSET, set a minimum score of 3 in each of the eight domains being assessed for the operator to be considered competent for the technical portion of the procedure. In another study, Dwyer et al. [59] added a criterion that participants were considered competent if they achieved an ASSET score of 24 or greater, except for the criteria score by Koehler et al. The criterion has not been demonstrated to reflect competency.

GRS also have a significant limitation in that experts are required for the evaluation process. Thus, a specific training protocol is necessary for evaluators to improve inter-rater reliability [58], limiting the generalizability of GRS.

Various GRS are used in arthroscopic skills training, but there is limited evidence on whether there is a superior scale for the assessment. Middleton et al. [60] compared three GRS to assess simulated arthroscopic skills and found that none demonstrated superiority, although ASSET had the highest frequency of use (10 of 17 studies) and has been validated in many studies of joints such as the knee [39,61], shoulder [62], hip[63], ankle [33] and wrist [64]. Among all GRS, only ASSET has demonstrated reliability in both simulated and clinical environments [15]. More studies are needed to consider ASSET as a promising assessment tool.

Because most operations in the clinical environment are complex and require high accuracy, checklists and GRS are selected as the evaluation method in most studies. Moreover, many do not use scales alone but also combine subjective and objective assessment tools that are suitable for assessing both basic skills such as diagnostic arthroscopy and advanced skills such as meniscectomy. This is a better way to evaluate the skill level of operators because it is based on multiple indicators.

## Limitations

There are several limitations to this review. Studies we included were heterogeneous in regard to study designs, methodology used and outcome measures. Given participants varied in experience level, assessing modality varied in context and skill, and different types of outcomes, objective and subjective measures were not directly comparable. This heterogeneity precluded a quantitative statistical analysis. Furthermore, the heterogeneity has also limited the scope for further analysis, such as determining whether the observed differences in reported outcomes were statistically significant. In addition, the majority of included studies focussed on the knee and the shoulder, while studies assessed other joints were limited and the evidence was insufficient. Moreover, although most studies combined objective metrics and subjective scales, there were no studies comparing them.

## Conclusion

Overall, both subjective and objective assessment tools can be used as feedback for basic arthroscopic skill training, but there are still differences in the frequency of application in different contexts. Despite this, combined use of subjective and objective assessment tools can be applied to more situations and skills and can be the optimal way for assessment.

## Data Availability

Not applicable. (The articles reviewed in this study are available in the public domain.)

## Appendix 1. Quality assessment of controlled intervention studies

**Table:**

Criteria	Yes	No	Other (CD,NR,NA)
1. Was the study described as randomized, a randomized trial, a randomized clinical trial, or an RCT?			
2. Was the method of randomization adequate (i.e. use of randomly generated assignment)?			
3. Was the treatment allocation concealed (so that assignments could not be predicted)?			
4. Were study participants and providers blinded to treatment group assignment?			
5. Were the people assessing the outcomes blinded to the participants' group assignments?			
6. Were the groups similar at baseline on important characteristics that could affect outcomes (e.g. demographics, risk factors, co-morbid conditions)?			
7. Was the overall drop-out rate from the study at endpoint 20% or lower of the number allocated to treatment?			
8. Was the differential drop-out rate (between treatment groups) at endpoint 15 percentage points or lower?			
9. Was there high adherence to the intervention protocols for each treatment group?			
10. Were other interventions avoided or similar in the groups (e.g. similar background treatments)?			
11. Were outcomes assessed using valid and reliable measures, implemented consistently across all study participants?			
12. Did the authors report that the sample size was sufficiently large to be able to detect a difference in the main outcome between groups with at least 80% power?			
13. Were outcomes reported or subgroups analysed prespecified (i.e. identified before analyses were conducted)?			
14. Were all randomized participants analysed in the group to which they were originally assigned, i.e. did they use an intention-to-treat analysis?			

Guidance for each question available at https://www.nhlbi.nih.gov/health-topics/study-quality-assessment-tools.

CD: cannot determine; NA: not applicable; NR: not reported.

## Appendix 2. Quality assessment tool for before-after (Pre-Post) Studies with no control group

**Table:**

Criteria	Yes	No	Other (CD,NR,NA)
1. Was the study question or objective clearly stated?			
2. Were eligibility/selection criteria for the study population prespecified and clearly described?			
3. Were the participants in the study representative of those who would be eligible for the test/service/intervention in the general or clinical population of interest?			
4. Were all eligible participants that met the prespecified entry criteria enrolled?			
5. Was the sample size sufficiently large to provide confidence in the findings?			
6. Was the test/service/intervention clearly described and delivered consistently across the study population?			
7. Were the outcome measures prespecified, clearly defined, valid, reliable, and assessed consistently across all study participants?			
8. Were the people assessing the outcomes blinded to the participants' exposures/interventions?			
9. Was the loss to follow-up after baseline 20% or less? Were those lost to follow-up accounted for in the analysis?			
10. Did the statistical methods examine changes in outcome measures from before to after the intervention? Were statistical tests done that provided *p* values for the pre-to-post changes?			
11. Were outcome measures of interest taken multiple times before the intervention and multiple times after the intervention (i.e. did they use an interrupted time-series design)?			
12. If the intervention was conducted at a group level (e.g. a whole hospital, a community, etc.) did the statistical analysis take into account the use of individual-level data to determine effects at the group level?			

Guidance for each question available at https://www.nhlbi.nih.gov/health-topics/study-quality-assessment-tools.

CD: cannot determine; NA: not applicable; NR: not reported.
